# First In Vitro Human Islet Assessment of Oleanolic Acid (OA) and Its Serine Conjugate: Enhanced Solubility with Comparable Effects

**DOI:** 10.3390/molecules30244716

**Published:** 2025-12-09

**Authors:** Runkai Yin, Li Zhao, Lina A. Huang, Rui Zhao, Andy Wu, Maggie Rao, Jason Wu, Claire Wu, Fouad R. Kandeel, Junfeng Li

**Affiliations:** 1 Department of Translational Research and Cellular Therapeutics, Beckman Research Institute of City of Hope, Duarte, CA 91010, USA; 2 Department of Gastroenterology, Hebei Children’s Hospital, No. 133 Jianhua South Street, Yuhua District, Shijiazhuang 050031, China

**Keywords:** oleanolic acid (OA), diabetes, islet transplantation, octanol-water partition coefficient, high-performance liquid chromatography (HPLC), hydrophilicity, human islet, viability, immunofluorescence staining

## Abstract

Oleanolic acid (OA) is a natural pentacyclic triterpenoid with reported hypoglycemic, hepatoprotective, antidiabetic, and anti-inflammatory activities. However, its limited aqueous solubility restricts its formulation and potential biomedical applications. To address this limitation, we designed a hydrophilic OA derivative, **1a**, by introducing an amino acid fragment at the C-28 position. We established a reverse-phase high-performance liquid chromatography (HPLC)-based method to measure the octanol–water partition coefficient (Log P*_ow_*) of OA and **1a**. Under neutral conditions, **1a** showed a markedly reduced Log P*_ow_* value (2.91 ± 0.02) compared with OA (4.30 ± 0.01), confirming substantially improved hydrophilicity. The biological compatibility of OA and **1a** was further evaluated using in vitro human islet cultures. Both compounds maintained high islet viability (approximately 90%). In addition, islets pre-treated with **1a** exhibited viability, purity, and insulin expression levels comparable to those observed with OA treatment, indicating that the C-28 modification preserved OA’s biological properties while improving solubility. Overall, this proof-of-concept study demonstrates that C-28 amino-functionalization can improve the physicochemical properties of OA without compromising its compatibility with human islets. The HPLC-based Log P*_ow_* method established here provides a practical analytical tool for future structure–activity investigations of OA derivatives, and the improved solubility of **1a** may facilitate its use in human islet preparation workflows.

## 1. Introduction

Islet transplantation presents a hopeful therapeutic strategy for managing diabetes by reinstating insulin secretion and achieving improved glycemic control [1]. It eliminates the requirement for external insulin injections, decreases the risk of severe hypoglycemia, and enhances the overall well-being of recipients [2]. Furthermore, transplanted islets can imitate natural insulin secretion in response to varying blood glucose levels, allowing for more precise glucose regulation than traditional therapies [3]. Nonetheless, a major obstacle to the success of islet transplantation is significant loss of islet mass in in vitro islet culture prior to transplantation. Isolated islets are highly vulnerable to damage during culture due to oxidative stress, nutrient deprivation, and exposure to pro-inflammatory conditions, leading to rapid deterioration in viability and functionality [4]. Prolonged culture periods further worsen islet deterioration, with reported declines in islet equivalents (IEQ) of up to 30% within the first 24 h [5]. These obstacles make it challenging to maintain sufficient islet mass and quality throughout the transplantation process. Additionally, the low survival rate of cultured islets prior to transplantation acts as a significant barrier to successful engraftment and long-term graft function.

**Figure 1:**
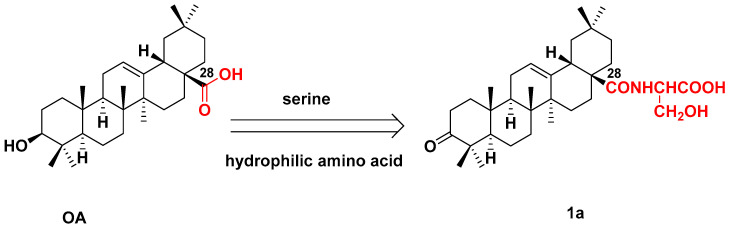
Chemical structure of OA and **1a**. The chemical structure of **1a** was derived from OA by introducing a fragment of serine, a hydrophilic amino acid, at the carbon-28 position. This modification was designed to enhance the hydrophilicity of OA, addressing its limited water solubility.

To address these limitations, optimizing in vitro culture conditions is critical, including the development of protective agents, such as oleanolic acid (OA, Figure 1) and its derivatives to enhance islet viability and function while mitigating oxidative and inflammatory damage. By improving pre-transplantation culture techniques, the efficacy and outcomes of islet transplantation can be significantly enhanced. OA, a pentacyclic triterpenoid extracted from a wide range of medicinal plants, is especially enriched in certain species such as olive trees (*Olea europaea*) and garlic (*Allium sativum*), and exhibits an excellent profile of non-toxicity and safety [6,7]. OA is extensively applied in the treatment of a broad range of diseases, such as cancer, diabetes, and gastrointestinal disorders, and is endowed with a vast array of advantageous pharmacological properties, such as antioxidant and anti-inflammatory effects [8,9,10,11,12]. Polycyclic triterpene compounds have been extensively studied for their potential anti-diabetic effects. For instance, Asiatic acid has demonstrated the ability to preserve islet beta cell mass and reduce hyperglycemia in STZ-induced diabetic mice [13]. Similarly, OA has shown promising results in diabetes mellitus treatment. OA exhibits multiple anti-diabetic effects, such as inhibiting glucose absorption in vitro, enhancing insulin secretion by activating beta cell M3 muscarinic receptors, and preserving islet beta cell mass in transplanted islets [14,15,16]. Its capacity to protect islet mass is attributed to its activation of the Nrf2 pathway, which shields islets from oxidative stress [17]. The accumulation of mitochondrial reactive oxygen species (ROS) is a key pathway that induces oxidative stress in beta cells. These cells are particularly vulnerable to oxidative damage due to their low levels of ROS-scavenging enzymes and proteins [18]. Oxidative stress within the islets leads to various forms of cellular damage, including DNA fragmentation, protein cross-linking, membrane phospholipid peroxidation, and the activation of inflammatory pathways [19]. This damage has been linked to both late-stage diabetic complications and the development of type 2 diabetes [18]. The Nrf2 pathway plays a crucial role by promoting the transcription of antioxidant enzymes, which can be significantly up-regulated by synthetic analogues of OA [19]. Additionally, OA has been shown to inhibit the NF-κB pathway, which prevents cell death by down-regulating the production of inflammatory cytokines [20]. The use of OA as an anti-diabetic agent is significantly enhanced by its effectiveness in improving beta-cell insulin response. OA, along with other triterpenoid acids, has been shown to inhibit the protein tyrosine phosphatase 1B (PTP-1B), a potent negative regulator of insulin production [21]. Furthermore, it has been found to increase the expression of Src-homology 2 domain-containing phosphotyrosine phosphatase (Shp-2), which plays a crucial role in facilitating insulin signaling [22]. Additionally, OA derivatives have demonstrated the ability to modulate peroxisome proliferator-activated receptors, which regulate genes involved in glucose homeostasis and lipid metabolism [23]. These findings indicate that OA and related compounds have significant potential in preserving beta-cell function and enhancing insulin response, making them promising candidates for further research in diabetes treatment.

Despite its potential therapeutic benefits, the clinical application of OA is severely limited by its poor water solubility of 4.61 mg/L (20 °C) [24]. Its low hydrophilicity is suggested to cause slow dissolution and limited penetration through gastrointestinal tissue, which further leads to very limited dose-independent oral bioavailability (0.7%) [25]. To improve the clinical application of OA, we intended to insert a hydrophilic amino acid fragment (serine) at the C-28 position to enhance hydrophilicity and generate a new derivative: **1a** (Figure 1). Existing research has shown that modifications at the C-28 position are a feasible strategy for OA functionalization [26,27,28,29,30,31]. In this study, we employed high-performance liquid chromatography (HPLC) to measure the octanol–water partition (P*_ow_*) values of OA and **1a** to verify the solubility enhancement achieved by this modification. Additionally, we examined and compared the effects of OA and **1a** in the pre-treatment of in vitro human islet cell cultures, aiming to provide greater flexibility in scheduling islet transplantation procedures. This work serves as a proof-of-concept evaluation to determine whether C-28 amino-functionalization can improve the physicochemical properties of OA while preserving its biological compatibility with human islets.

### Advantages of Choosing Human Islets Compared to Rodent Islets

Rodent pancreatic islets have long been the primary model for experimental research in islet isolation and transplantation studies [32]. However, despite their similarities in size distribution [33,34,35], human pancreatic islets possess unique characteristics that make them not only more representative for certain research applications but also more clinically relevant, particularly in the context of islet transplantation in human patients. First, the physiological structure of human and rodent islets differs substantially. The beta-to-alpha cell ratio in mouse islets is higher than in human islets [35]. Additionally, mouse islets are reported to have a distinct architecture, with a core almost exclusively comprising beta cells and a surrounding mantle of non-beta cells, whereas human islets exhibit more heterogeneous cellular patterns [36]. Human islets also demonstrate unique functional and regulatory properties. For instance, they have distinct distributions and functions of key regulators involved in glucose-stimulated insulin secretion [37]. Significant differences have been observed in the expression of antioxidant enzymes [37,38], glucose transporters [39], and the proliferative capacity of beta cells [40] between human and rodent islets. Moreover, G-protein-coupled receptors (GPCRs) exhibit marked differences between human and mouse islets. For example, G-protein-coupled receptor 44 (GPR44) is enriched more than threefold in human islets compared to mouse islets, highlighting functional divergences in signaling pathways [41].

Given these distinctions in architecture, function, and molecular expression, human islets provide a more physiologically relevant model for studies that aim to inform human clinical applications. Therefore, human islets were chosen for this in vitro study to ensure the generation of reliable data for future in vivo islet transplantation experiments.

## 2. Results and Discussion

### 2.1. Measurement of Log P_ow_ Values for OA and ***1a***

Log P*_ow_* is a critical parameter for understanding the balance between a compound’s hydrophilicity and lipophilicity using a two-phase system of *n*-octanol and water [42]. The octanol–water partition ratio offers an approximate measure of how chemicals distribute between aqueous and organic phases, providing insights into their physicochemical properties [43]. A higher Log P*_ow_* value indicates a hydrophobic profile, while a lower value suggests hydrophilicity. These values can be determined experimentally or predicted using commercially available software. The balance between hydrophilicity and lipophilicity is crucial in drug design and selection, as extreme hydrophilicity or lipophilicity can limit a drug’s effectiveness. Highly hydrophilic compounds may face challenges with membrane permeability, while excessively lipophilic drugs might exhibit poor dissolution rates, both leading to suboptimal absorption [44]. Achieving a suitable Log P*_ow_* value is vital for optimizing a drug’s pharmacokinetic and pharmacodynamic profiles. This balance depends on multiple factors, including the target organ, route of administration, and therapeutic objectives. The octanol–water partitioning system serves as an essential tool in evaluating and refining these parameters to meet specific therapeutic goals [45].

Currently, several experimental methods are available for measuring Log P*_ow_*, including the shake flask method, slow stirring method, and HPLC method [46,47]. The shake flask method is a simple, cost-effective approach that is easy to parallelize. However, it has limitations such as low reproducibility and limited detailed insights into culture performance [48]. The slow stirring method, on the other hand, is highly accurate and precise, making it suitable for determining the Log P*_ow_* of highly hydrophobic chemicals. Nevertheless, this method often requires sensitive analytical techniques to analyze the water phase effectively [46,49]. In contrast, HPLC has become a popular choice in scientific research and is widely accepted across various fields. It offers several advantages, including greater efficiency, shorter determination times compared to traditional methods [50], and high sensitivity, enabling accurate results even with small sample volumes [51]. In this study, the HPLC method was selected to evaluate the hydrophilicity of modified OA due to its efficiency and precision.

In this study, **1a**, a newly synthesized compound, required validation of feasible HPLC conditions. A 40 mg/L standard sample of **1a** was analyzed in a pilot experiment to ensure that all HPLC parameters were properly set. It was observed that **1a** exhibited a similar retention time and UV absorption wavelength to OA, confirming the feasibility of the HPLC conditions. Establishing standard calibration curves was a critical step to ensure reliable and high-quality results. A stock solution concentration of 400 mg/L was employed, with the concentrations of compounds in the organic phase of equilibrium solutions being lower than 400 mg/L. Consequently, a series of calibration standards were prepared, ranging from 2.5 mg/L to 120 mg/L with increasing concentration spacing.

**Figure 2:**
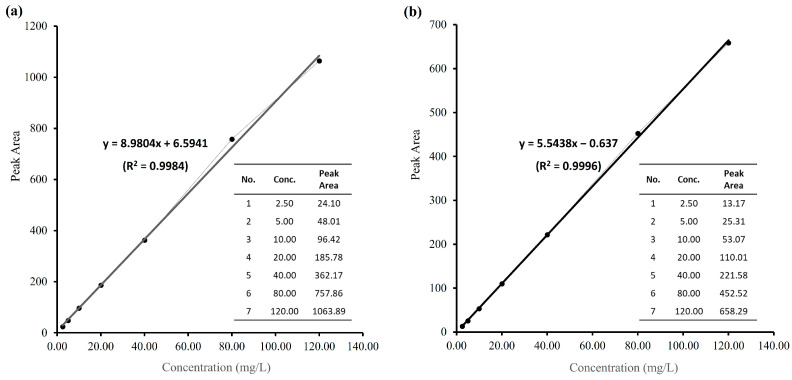
Calibration curves and peak area values for OA and **1a**. (**a**) Calibration curve for OA: The curve was generated using a concentration range of 2.5 to 120 mg/L, achieving a high correlation coefficient (R^2^ = 0.9984). (**b**) Calibration curve for **1a**: The curve was generated using a concentration range of 2.5 to 120 mg/L, achieving an exceptionally high correlation coefficient (R^2^ = 0.9996).

For the linear regression equation, the correlation coefficient (R^2^) was the primary criterion used to assess the reliability of the regression analysis [52]. In a previous study, Pagliano and Meija demonstrated that a calibration equation with an R^2^ value exceeding 0.99 indicated a strong linear relationship [53]. In this research, the linear regression analysis for OA produced the equation:

y = 8.9804x + 6.5941″, with R^2^ of 0.9984 (Figure 2a).

For **1a**, the calibration curve was also linear with the equation:

y = 5.5438x − 0.637″ and R^2^ of 0.9996 (Figure 2b).

Both compounds exhibited R^2^ values exceeding 0.99, indicating strong fits of the data to the linear models. Notably, **1a** showed an extremely high R^2^ value, suggesting a nearly perfect linear relationship.

We determined the Log P*_ow_* values for OA and **1a**. Notably, the experimental Log P*_ow_* value for **1a** in water was significantly reduced (2.91 ± 0.02) compared to OA (4.30 ± 0.01), indicating increased aqueous solubility for **1a**. To further support this observation, we calculated the log *p* values using ChemDraw 19.0. The predicted log *p* for OA was 7.47, while that for **1a** was 6.45, showing a notable reduction in lipophilicity due to structural modification at the C-28 position. Although these values are not experimentally derived, ChemDraw-calculated log *p* has been reported as a feasible computational approach for assessing relative hydrophilicity. The lower predicted log *p* of **1a** further supports the hypothesis that the introduction of a hydrophilic amino acid moiety at C-28 enhances aqueous solubility. Published data indicate that online software platforms predict a wide range of log *p* values for oleanolic acid, as reported in the study “UV-Spectrophotometric Determination of Partition Coefficient (log p) of Oleanolic Acid and Its Comparison with Software Predicted Values.” The authors noted that, according to SwissADME (Swiss Institute of Bioinformatics, SwissADME. Available at: http://www.swissadme.ch), the predicted log *p* values for oleanolic acid fall within the range of 3.94 to 7.49 [54]. This variation is possibly attributable to differences in algorithm type, ionization assumptions, and molecular fragment libraries. Therefore, we used a single consistent method (ChemDraw 19.0) to calculate the theoretical log *p* values of OA and **1a**, focusing on the relative trend between the two compounds rather than the absolute predicted log *p* value. Importantly, the actual hydrophobicity of both compounds was experimentally determined using our HPLC-based log P*_ow_* method, providing a more reliable and reproducible assessment than computational predictions. In addition, we conducted a comparative solubility test in phosphate-buffered saline (PBS, pH 7.4). Compound **1a** showed good solubility in PBS, while OA was insoluble under the same conditions. These results collectively support the conclusion that modification at the C-28 position improves aqueous solubility.

### 2.2. Evaluation for Human Islets Cultured with OA and Its Derivative ***1a***

#### 2.2.1. OA/**1a** Culture Concentration and Culture Period

This concentration of treatment was carefully selected to balance efficacy and safety, ensuring minimal cytotoxicity to the islets while providing sufficient exposure to observe potential protective effects. Previous in vitro studies investigating the anti-inflammatory properties of OA have employed concentrations ranging from 500 nM to 10,000 nM [55]. Notably, cytotoxicity assays on cell lines such as human hepatoma (HepG2), human epithelial colorectal adenocarcinoma (Caco-2), and human retinoblastoma (Y-79) demonstrated significant reductions in cell viability at concentrations of 16 µM, 2 µM, and above, respectively [56]. However, the sensitivity of isolated human islets to OA remains unclear. To minimize the risk of cytotoxicity, particularly given the limited availability of human islets, we opted for an initial concentration of 600 nM (0.6 µM) for both OA (group 1) and **1a** (group 2). **1a**, as a novel OA derivative, was expected to exhibit pharmacological properties similar to OA, except for improved hydrophilicity. Both compounds were prepared and compared in DMSO due to the limited water solubility of OA. A stock solution (0.2 mg in 0.6 mL DMSO) was prepared and subsequently diluted into the culture media to achieve the desired concentration. For each treatment, the added DMSO volume was 1.6 µL per 2 mL of culture medium, and the same DMSO volume was added to the DMSO control group to ensure consistency across all wells. The DMSO concentration in all wells was maintained below 0.1% to avoid potential toxicity to the islets [57].

While isolated islets are known to deteriorate rapidly in culture, with a reporting up to 30% decrease in islet equivalent (IEQ), a standardized measured based on the volume of a spherical islet with a diameter of 150 µm, after overnight culture [5], this extended period was deemed essential for assessing the protective effects of the compounds. In clinical allogeneic islet transplantation, islets are typically cultured for 48–72 h while recipients undergo immunosuppression [58]. In experimental settings, culture periods are usually shorter, around 20 h [5]. However, to capture the long-term impacts of OA and **1a** on islet survival and function, a longer culture period was required. The chosen two-week timeframe allowed for a comprehensive evaluation of whether OA and **1a** could mitigate the inherent vulnerability of islets to cell loss and deterioration during extended culture, providing valuable insights into their potential therapeutic effects.

#### 2.2.2. Viability Evaluation for Human Islets Cultured with OA and Its Derivative **1a**

**Figure 3:**
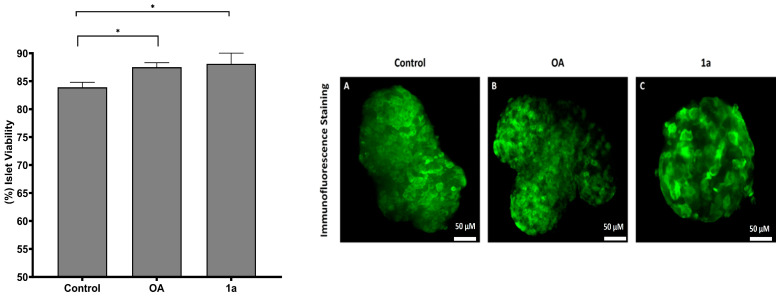
Comparison of islet viability and insulin expression levels after two weeks in culture. (**Left**) Viability was quantified based on FDA (green, viable cells) and PI (red, dead cells) fluorescence using CellSens imaging software (version 1.12, Olympus). Error bars represent standard deviation. Islets treated with OA and its derivative demonstrated favorable viability values (83.9% for DMSO; 88.1% for **1a**; 87.5% for OA). (**Right**) Immunofluorescence staining for insulin (green) in cultured islets following two weeks in culture. Scale bar, 50 µm. (**A**): Islet sample in DMSO control. (**B**): Islet sample from the OA treatment group. (**C**): Islet sample from the **1a** treatment group. Both islet samples displayed similar degrees of insulin expression, indicating preserved functional activity. One asterisk (*) indicates *p* value smaller than 0.05 (*p* < 0.05). Statistical significance was defined as *p* < 0.05.

In this study, the viability of cultured human islets was evaluated using the fluorescein diacetate/propidium iodide (FDA/PI) staining method, a widely validated tool for assessing islet quality [59,60]. This method differentiates live and dead cells based on fluorescence: living islet cells convert non-fluorescent FDA into green fluorescent fluorescein, while dead cells exhibit red fluorescence due to PI penetration into permeabilized cell membranes [61]. Viable cells with intact membranes appear as bright green fluorescent areas under a microscope. The viability results showed that DMSO control group exhibited 83.9% viability, whereas both OA- and **1a**-treated islets maintained viability levels above 87% (Figure 3). The findings indicate that OA improved human islets survival during in vitro culture, consistent with its previously reported anti-inflammatory and antioxidant potentials. For example, OA-treated islets transplanted under the kidney capsules of streptozotocin-induced diabetic mice demonstrated prolonged graft survival, up to 23 ± 3 d [16]. Despite these known benefits, OA’s effects on human islets prior to transplantation have rarely been examined. Importantly, islets treated with **1a** showed a viability of 88.1%, comparable to that of the OA-treated group (87.5%). This suggests that the hydrophilic modification at the C-28 position preserved the biological compatibility of OA with human islets. Although **1a** did not markedly exceed OA in viability enhancement, it maintained OA’s protective properties while providing the added advantage of significantly improved aqueous solubility, supporting its potential utility in isolated islet preparation workflows.

#### 2.2.3. Immunofluorescence Staining Assay for Post-Cultured Human Islets

The primary function of human islets is to secrete insulin, which regulated blood glucose levels. After two weeks of culture, the islet samples were subjected to insulin immunofluorescence staining to assess insulin expression, a key indicator of functional integrity. Immunofluorescence is a widely used method for visualizing cell-specific protein expression and provides essential insights into islet functionality. Fixation is a crucial preliminary step in immunofluorescence staining to prevent autolysis, mitigate putrefaction, and preserve cellular morphology [62]. While no universal fixative works for all antigens, formalin, a cross-linking fixative, was chosen for this study due to its widespread acceptance and reliability [63]. The indirect method of immunofluorescence staining was employed, involving a two-step process: Primary Antibody Binding: An insulin-specific primary antibody binds to the target epitope. Secondary Antibody Binding: A fluorophore-tagged secondary antibody binds to the primary antibody, enabling fluorescence detection. This indirect method is preferred for its high sensitivity and superior detection capability compared to the direct method, where the fluorophore is directly conjugated to the primary antibody [64]. Representative images of insulin immunofluorescence staining are shown in Figure 3 for the OA and **1a** treatment groups. In all samples, insulin-positive staining (green fluorescence) was densely distributed throughout the islets. This consistent expression pattern indicates robust insulin production and demonstrates that two-week pre-treatment with OA or **1a** did not impair insulin expression in cultured human islets.

#### 2.2.4. Dithizone (DTZ) Staining Assay for Post-Cultured Human Islets

**Figure 4:**
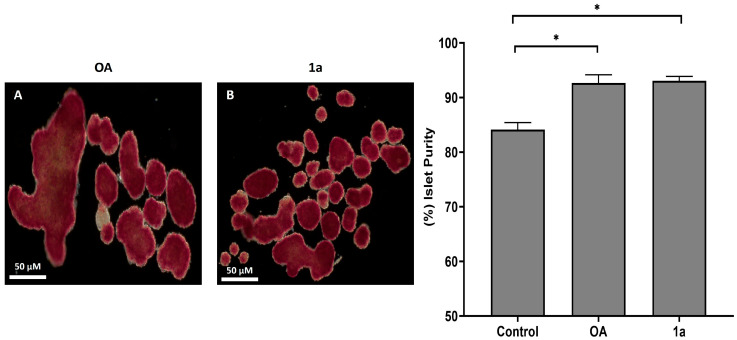
DTZ staining assay for post-cultured human islets. (**Left**) Representative DTZ-stained islet samples from each group, with zinc-rich beta cells appearing red. (**A**): OA treatment group; (**B**): **1a** treatment group. Scale bar, 50 µm. (**Right**) Quantitative comparison of islet purity levels after two weeks in culture. Purity levels were 84.1% for islets control, 92.7% for islets co-cultured with OA, and 93.1% for islets co-cultured with **1a**. Error bars represent standard deviation. One asterisk (*) indicates *p* value smaller than 0.05 (*p* < 0.05). Statistical significance was defined as *p* < 0.05.

We evaluated the purity of post-cultured human islets, as islet purity significantly affects transplantation efficacy and safety. Islet purity can decline during in vitro culture due to factors such as limited oxygen and nutrient availability [65,66]. DTZ staining, a widely accepted technique, is frequently used to determine islet purity by selectively staining pancreatic beta cells, which contain high zinc (Zn^2+^) levels [67,68]. DTZ acts as a zinc-chelating agent, staining high-zinc cells bright red (DTZ-positive structures) while leaving other cells unstained [69]. So, islet purity was assessed using a novel iDTZ formulation. Representative DTZ-stained images of islet samples are shown in Figure 4 and the DTZ-positive structures are labeled in purple, representing the stained islet areas. The corresponding purity levels of cultured islets, as calculated through software analysis, were 92.7% for the OA pre-treatment group and 93.1% for the **1a** pre-treatment group, both showing a significant enhancement compared to the DMSO control group (84.1%).

These observations suggest that both OA and **1a** effectively preserved islet morphology and reduced contamination from non-islet tissue over the two-week culture period. Although the starting islet purity was comparable among all groups, the elevated final purity in the OA- and **1a**-treated groups suggests that these compounds helped maintain islet structural integrity and viability during extended in vitro culture. Since islet purity is an important determinant of transplantation efficiency and safety, the ability of both compounds to sustain higher purity underscores their potential value in islet preparation workflows. Importantly, **1a** demonstrated purity levels comparable to those of OA, indicating that the C-28 hydrophilic modification preserved OA’s biological compatibility with human islets. While **1a** did not produce a substantially greater effect than OA in this assay, it offers a significant practical advantage: unlike OA, which requires organic solvents for dissolution, **1a** readily dissolves in aqueous buffers. This improved solubility may facilitate more convenient formulation and handling during islet pre-treatment. Additional studies on the solubility-enhanced formulation of **1a** and its in vitro and in vivo biological performance will be presented in a subsequent publication.

## 3. Materials and Methods

### 3.1. Materials

OA, 1-Octanol (99%), methanol, and acetonitrile were procured from Thermo Fisher Scientific. Dimethyl sulfoxide (DMSO), dithizone (DTZ), ammonium hydroxide solution, fluorescein diacetate (FDA), and propidium iodide (PI) were obtained from Sigma Aldrich (Saint Louis, CA, USA). Milli-Q water (MQH_2_O; pH 7) was utilized for all experiments.

HPLC was performed on an Agilent 1260 Infinity II System (Agilent Technologies, Santa Clara, CA, USA) equipped with a quaternary pump and degasser, an automated sample injector, a column compartment, and a variable-wavelength UV detector. The analytical column used was Agilent, Luna_C18, 4 µm, 150 × 4.6 mm; Heracell^TM^ 150c CO_2_ incubator was from Thermo Fisher Scientific (Waltham, MA, USA); fluorescence microscope (BZ-X700 Series; Osaka, Japan) was from Keyence Corporation. CellSens imaging software was from Olympus (Tokyo, Japan).

### 3.2. Methods

#### 3.2.1. Synthesis of **1a**

**Scheme 1:**
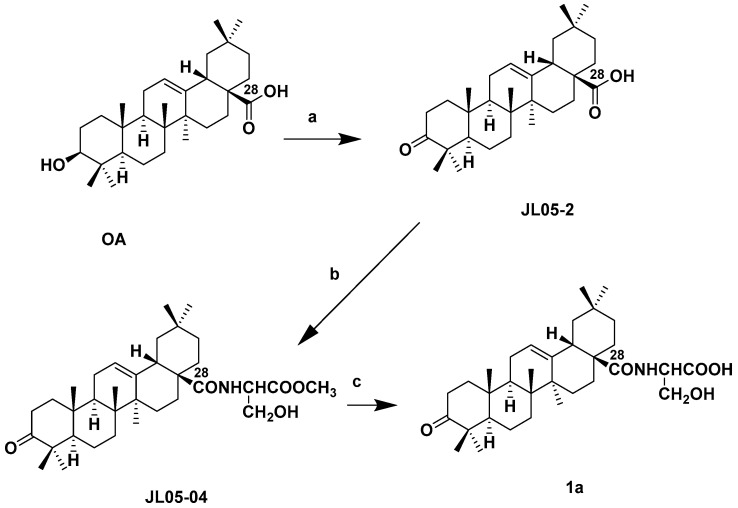
Synthesis of compound **1a** from OA. Reagents and conditions: (a). Jones oxidation; (b). Oxalyl chloride, dry CH_2_Cl_2_, rt, 24 h, then, serine methyl ester, triethylamine, rt, 6 h. (c). 4 M NaOH, 40 °C, 3.5 h.

**1a** was prepared following literature methods with minor modifications (Scheme 1) [28,70,71]. To a solution of OA (1) (4.56 g, 10 mmol) in CH_2_Cl_2_/Acetone (1:1, 300 mL) in an ice bath was added Jones reagent dropwise until the color of the solution changed from orange to green, then stirred at room temperature for 15 min. To the reaction mixture i-PrOH (3 mL) and water (12 mL) were added, and the resulting mixture was stirred at room temperature for 10 min. Water (90 mL) and CH_2_Cl_2_ (60 mL) were added to the mixture and organic layer was separated, which was then washed with saturated aq. NaCl (60 mL), dried, and concentrated in vacuo. 3-Oxooleanolic acid was obtained by crystallisation in methanol. Yield was 90%. ^1^H NMR (700 MHz, CDCl_3_) δ 5.29 (s, 1H), 2.83 (dd, 1H), 2.53 (m, 1H), 2.36 (m, 1H), 1.15 (s, 3H, CH_3_), 1.09 (s, 3H, CH_3_), 1.04 (s, 3H, CH_3_), 1.03 (s, 3H, CH_3_), 0.95 (s, 3H, CH_3_), 0.92 (s, 3H, CH_3_), 0.80 (s, 3H, CH_3_).

A solution of JF05-02 (0.998 mmol, 0.44 g) in dry CH_2_Cl_2_ (20 mL) was treated with oxalyl chloride (0.50 mL) in an ice bath and stirred at room temperature for 24 h. The reaction mixture was concentrated under reduced pressure, and the residue was treated with cyclohexane (2 × 10 mL) and reconcentrated to give the crude acyl chloride intermediate. The intermediate was dissolved in dry CH_2_Cl_2_ (30 mL), followed by addition of L-serine methyl ester hydrochloride (2.77 mmol, 0.476 g) and triethylamine (0.4 mL). The mixture was stirred at room temperature for 6 h, then partitioned with water (9 mL) and adjusted to pH 3 with 2 N HCl. The organic layer was separated and concentrated under reduced pressure to give a white solid. The solid was collected by filtration, washed with water until the washings reached pH 6, and dried. Purification by silica gel chromatography (hexane/ethyl acetate = 3:1) afforded JF05-04 as a white powder. JF05-04 (0.450 mmol, 0.25 g) was dissolved in CH_3_OH–THF (1:1.5, 16 mL) and treated with 4 M NaOH (4 mL). The mixture was stirred at 40 °C for 3.5 h, and the solvents were removed in vacuo. The residue was suspended in water and adjusted to pH 3 with 2 N HCl. Removal of the organic solvents under reduced pressure produced a white solid, which was filtered, washed with water to pH 6, and dried to afford the final product as a white powder. Yield: 88.0%; ^1^H NMR (700 MHz, CDCl_3_): 7.09 (d, 1H), 5.52 (s, 1H), 4.40 (m, 1H), 4.20 (m, 1H), 3. 79 (m, 1H), 2.65–2.52 (m, 2H), 2.42 (m, 1H), 1.19 (s, 3H, CH_3_), 1.09 (s, 3H, CH_3_), 1.04 (s, 3H, CH_3_), 1.03 (s, 3H, CH_3_), 0.92 (s, 3H, CH_3_), 0.91 (s, 3H, CH_3_), 0.78 (s, 3H, CH_3_).

#### 3.2.2. Measuring Log P*_ow_* by HPLC

##### HPLC Program Conditions

The HPLC analysis was performed using acetonitrile (ACN) (mobile phase A) and 0.3% phosphoric acid (mobile phase B) at a volumetric ratio of 85:15. The UV detector was set at 210 nm, with an injection volume of 20 µL. The column temperature was maintained at 30 °C, with a flow rate of 1 mL/min and a total run time of 10 min.

##### Standard Calibration Curves of OA and **1a**

OA was dissolved in methanol to prepare a stock solution with a concentration of 400 mg/L. The stock solution was then diluted quantitatively with methanol to prepare calibration standards at sequential concentrations of 120, 80, 40, 20, 10, 5, and 2.5 mg/L. Each standard solution (2.5, 5, 10, 20, 40, 80, and 120 mg/L) was analyzed sequentially using HPLC. A calibration curve was plotted based on the concentrations and their corresponding relative peak area values. The regression equation for the calibration curve was generated accordingly. Similarly, a standard calibration curve for compound **1a**, along with its regression equation, was developed using the same procedure.

##### Sample Preparation and Partitioning Procedure

Mutually saturated solutions of 1-octanol and water (pH 7) were prepared by vigorously shaking equal amounts of water and 1-octanol in a separating funnel, followed by overnight settling.

**Figure 5:**
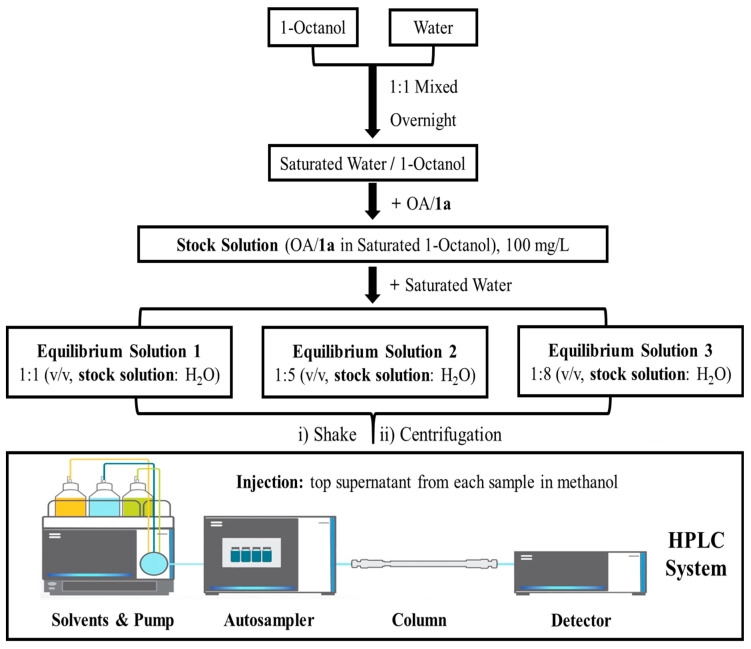
Process diagram illustrating the preparation of equilibrium solutions for OA/**1a** with water (pH 7) and 1-octanol.

OA was dissolved in the saturated 1-octanol solutions (prepared above) to create stock solutions with a concentration of 100 mg/L. These stock solutions were mixed with saturated water in varying volume ratios (stock solution: water) to produce three equilibrium solutions: Equilibrium solution 1: *v*/*v* = 1:1; Equilibrium solution 2: *v*/*v* = 1:5 and Equilibrium solution 3: *v*/*v* = 1:8. Each equilibrium solution was shaken at 37 °C and 200 rpm for 24 h, then centrifuged at 23 °C and 1200 rpm for 5 min. From each equilibrium solution, 50 µL of the top supernatant was diluted with 150 µL of methanol, and the resulting mixtures were analyzed via HPLC to determine the corresponding peak area values. The same procedure was applied to analyze the peak area values for compound **1a**. A detailed flowchart of this procedure is presented in Figure 5.

##### Calculation of Log P*_ow_* Value

The peak area value (variable y_1_) from the equilibrium solution 1 sample (*v*/*v*: 1:1) was input into the corresponding regression equation to calculate the concentration value x_1_. Similarly, the peak area value (variable y_2_) from the equilibrium solution 2 or 3 sample (*v*/*v*: 1:5 or 1:8) was used with the regression equation to calculate the concentration value x_2_. The Log P*_ow_* value was calculated using the following formula:

$$\mathrm{Log}\;P_{ow}=\mathrm{Log}\frac{x_{2}}{\left(x_{1}-x_{2}\right)\times r}$$

Here, r refers to the volume ratio of the equilibrium solutions (1:5 or 1:8).

#### 3.2.3. OA and **1a** Pre-Treatment

##### OA/**1a** Culture Concentration and Culture Period

Human islets were treated with OA and its derivative, **1a** (0.2 mg/0.6 mL stock in DMSO), at a final concentration of 600 nM during the culture period. Control islets received an equivalent volume of dimethyl sulfoxide (DMSO; Sigma-Aldrich, St. Louis, MO, USA) in culture medium. A two-week culture period was selected to thoroughly evaluate the effects of OA and **1a** pre-treatments on islet viability and function.

##### Human Islet Culture

Isolated human islet samples were cultured in a 6-well plate at 150 IEQ/well across three groups: Group 1: DMSO control; Group 2: OA dissolved in DMSO; Group 3: **1a** dissolved in DMSO. Each group consisted of six replicates (n = 6/group) with a final treatment concentration of 600 nM. Islets were maintained in CMRL 1066 media (2 mL inside the insert and 2 mL outside the insert) supplemented with 0.01% insulin-like growth factor-1, 1% heparin, and 2% human serum albumin. Cultures were incubated at 37 °C in a CO_2_ incubator for 14 d, with the media refreshed every 3 d.

##### In Vitro Islet Viability Assay

The viability of cultured human islets was assessed after 2 weeks using FDA/ PI staining. The FDA/PI working solution was prepared in a dark room by dissolving: 5 µL FDA solution (48 µM; FDA 2 mg dissolved in 100 mL acetone) and 5 µL PI solution (1.5 mM; commercially available concentration). Both were diluted into 490 µL PBS, yielding final concentrations of 0.48 µM FDA and 15 µM PI. Human islets were stained with the FDA/PI solution for 5 min at room temperature and then washed with PBS. Stained islets were transferred to a plate for imaging, and fluorescence images were captured and analyzed using CellSens imaging software.

Viability calculation formula:

Overall viability (%) = 100 − (PI-positive area/islet area) × 100.

##### DTZ Staining for Islet Purity

The iDTZ solution was prepared by dissolving 80 mg of DTZ in a solution containing: 62% DMSO, 37.5% methanol and 0.5% ammonium hydroxide solution (*v*/*v*/*v*). A 100 µL islet sample was pipetted into 250 µL of iDTZ solution in an islet cell counter (ICC)-compatible dish. The dish was gently agitated and placed on the imaging stage at 22 °C. The stained areas were analyzed using CellSens imaging software, which identified iDTZ-positive structures.

##### Immunofluorescence Staining

Post-cultured islet samples were fixed in formalin for 30 min and washed with PBS for 30 min. They were then permeabilized with 0.2% Triton X-100 (MilliporeSigma, Burlington, VT, USA), and blocked with 2.5% goat serum for 2 h at room temperature. Subsequently, the islets were incubated overnight at 4 °C with a primary insulin antibody (1:1000 dilution). After washing with PBS, samples were treated with a secondary antibody (1:1000 dilution) for 30 min in a dark room at room temperature. Counterstaining with 4′,6-diamidino-2-phenylindole (DAPI) followed, and the samples were mounted on microscope slides and dried overnight in a dark room at room temperature.

## 4. Conclusions

In this study, we generated a hydrophilic derivative of OA, designated as **1a**, by introducing a polar fragment at the C-28 position. A practical HPLC-based method was established to quantify the Log P*_ow_* values of OA and **1a**, confirming that the C-28 modification substantially improved aqueous solubility and enabled dissolution in buffer without the need for organic solvents. Evaluation in human islet cultures demonstrated that **1a** maintained islet viability and purity at levels comparable to OA, indicating that its enhanced hydrophilicity did not compromise the biological compatibility of OA with human islets. These findings support the feasibility of C-28 amino-functionalization as a strategy to improve the physicochemical properties of OA while preserving its in vitro protective profile.

This work provides a proof-of-concept foundation for future development of OA derivatives with optimized solubility and formulation characteristics. Ongoing studies using an islet transplantation mouse model will further investigate the biological performance of **1a**, and expanded analyses of solubility-enhanced OA derivatives will be reported in subsequent publications.

## Data Availability

Data are contained within the article.
